# Cardiopulmonary interactions during ventilator weaning

**DOI:** 10.3389/fphys.2023.1275100

**Published:** 2023-09-07

**Authors:** Philippe Vignon

**Affiliations:** ^1^ Medical-surgical ICU and Inserm CIC 1435, Dupuytren University Hospital, Limoges, France; ^2^ Faculty of Medicine, University of Limoges, Limoges, France

**Keywords:** weaning, mechanical ventilation, heart-lung interactions, pulmonary edema, echocardiography

## Abstract

Weaning a critically-ill patient from the ventilator is a crucial step in global management. This manuscript details physiological changes induced by altered heart-lung interactions during the weaning process, illustrates the main mechanisms which could lead to weaning failure of cardiac origin, and discuss a tailored management based on the monitoring of changes in central hemodynamics during weaning. The transition from positive-pressure ventilation to spontaneous breathing results in abrupt hemodynamic and metabolic changes secondary to rapidly modified heart-lung interactions, sudden changes in cardiac loading conditions, and increased oxygen demand. These modifications may elicit an excessive burden on both the respiratory and cardiovascular systems, result in a rapid and marked increase of left ventricular filling pressure, and ultimately result in a weaning-induced pulmonary oedema (WIPO). The T-piece trial induces the greatest burden on respiratory and cardiocirculatory function when compared to spontaneous breathing trial using pressure support ventilation with positive or zero end-expiratory pressure. Since LV overload is the mainstay of WIPO, positive fluid balance and SBT-induced acute hypertension are the most frequently reported mechanisms of weaning failure of cardiac origin. Although the diagnosis of WIPO historically relied on an abrupt elevation of pulmonary artery occlusion pressure measured during right heart catheterization, it is nowadays commonly documented by echocardiography Doppler. This non-invasive approach is best suited for identifying high-risk patients, depicting the origin of WIPO, and tailoring individual management. Whether this strategy increases the success rate of weaning needs to be evaluated in a population at high risk of weaning failure of cardiac origin.

## Introduction

Weaning a critically-ill patient from the ventilator is a challenging process and a key step in overall therapeutic management since weaning failure is associated with a prolonged duration of mechanical ventilation and length of stay in the intensive care unit (ICU) (Epstein et al., 1997; Thille et al., 2013). In turn, prolonged invasive mechanical ventilation is associated with ICU morbidity and mortality (Boles et al., 2007; Frutos-Vivar et al., 2011; Beduneau et al., 2017). Weaning failure is multifactorial and its rate is approximately two-fold in high-risk patients (e.g., age >65 years, chronic heart or respiratory disease) when compared to other patients (≈20% vs. ≈10%) (Thille et al., 2019; Goudelin et al., 2020).

Weaning from positive-pressure ventilation has been compared to an exercise stress test for the cardiovascular system (Pinsky, 2000). Indeed, spontaneous breathing trial (SBT) induces abrupt hemodynamic changes and increases the work of breathing, which may ultimately result in increased left ventricular (LV) filling pressure and subsequent weaning-induced (hydrostatic) pulmonary oedema (WiPO) (Teboul et al., 2010; Liu et al., 2016). Accordingly, inadequate cardiovascular response is one of the leading mechanisms of SBT or extubation failure (Pinsky, 2000; Boles et al., 2007).

Historically, the diagnosis of WIPO relied on an abrupt elevation of pulmonary artery occlusion pressure (PAOP) during right heart catheterization (Lemaire et al., 1988). Currently, critical care echocardiography (CCE) plays an increasing role in both the diagnosis and management of acute respiratory failure, since it is non-invasive, easy to perform at the bedside, and provides valuable anatomical and hemodynamic information in real-time (Vignon et al., 2016). When associated with lung and respiratory muscle ultrasound, it further refines diagnostic information on the leading mechanism of respiratory compromise (Bataille et al., 2014; Mayo et al., 2016; Llamas-Alvarez et al., 2017; Tuinman et al., 2020). This manuscript aims at illustrating main underlying mechanisms of WIPO and discussing tailored management based on the monitoring of changes in central hemodynamics resulting from modified heart-lung interactions during SBT.

### Hemodynamic changes induced by SBT

Interruption of positive-pressure ventilation increases venous return, LV afterload and the work of breathing. Greater respiratory muscle oxygen consumption and cardiac workload may induce heart ischemia and decreased LV compliance (Perren and Brochard, 2013; Teboul, 2014). Overall, these intricate phenomena contribute to an abrupt increase of LV filling pressure, and may ultimately result in WIPO (Lemaire et al., 1988). The clinical consequence of the cardiovascular stress induced by SBT is more pronounced in patients with reduced cardiac reserve and volume overload (Pinsky, 2000). Noticeably, additional cardiovascular workload generated by SBT can be anticipated in patients with severe obstructive and restrictive pulmonary diseases, since hemodynamic alterations are emphasized by the larger negative pleural swings during inspiratory efforts (Perren and Brochard, 2013). In COPD patients, right ventricular (RV) afterload may also increase secondary to worsening hypoxemia, hypercapnia and intrinsic positive end-expiratory pressure, and result in RV dilatation. This in turn impedes LV filling due to ventricular interaction and participates in deleterious hemodynamic changes during SBT (Teboul, 2014).

The transition from a positive intrathoracic pressure under mechanical ventilation to a negative pleural pressure generated by the respiratory muscles accounts for the consistent rise of PAOP during SBT (Lemaire et al., 1988; Teboul et al., 1988). Previous studies showed that the magnitude of mean PAOP elevation was markedly larger in patients who developed a WIPO during SBT (8 ± 5 mmHg to 25 ± 13 mmHg) (Lemaire et al., 1988), when compared to that measured in patients with successful SBT (10 ± 2 mmHg to 14 ± 3 mmHg) (Teboul et al., 1988). In 19 ventilated medical and surgical patients, Jubran et al. (Jubran et al., 1998) reported that all patients who failed SBT had a PAOP >18 mmHg at the end of the procedure (27 ± 5 mmHg), whereas all patients who succeeded SBT did not (9 ± 2 mmHg). Finally, the incidence rate of SBT-induced PAOP elevation is high in difficult-to-wean patients who failed at least one SBT (Cabello et al., 2010). Overall, this underlines the pivotal role of increased LV filling pressure among the various components which ultimately lead to SBT failure (Lemaire et al., 1988).

### Clinical settings

#### Definition of weaning and timing of WIPO

Among patients undergoing ventilator weaning, three groups have been distinguished by an international consensus conference (Boles et al., 2007): simple weaning defining patients undergoing a successful process from initiation of SBT to extubation on the first attempt; difficult weaning denoting patients who fail initial SBT and require up to three SBTs or as long as 7 days from the first SBT to achieve successful extubation; prolonged weaning referring to patients who fail at least three SBTs or require more than 7 days after the first SBT to be successfully weaned from the ventilator. More recently, the WIND criteria defined the start of weaning as the first separation attempt by any type of SBT, with or without extubation, or by a planned or unplanned extubation directly performed without prior SBT (Beduneau et al., 2017). Four mutually exclusive groups of patients were then defined, with distinct mortality risk: no weaning (no separation attempt possible), short weaning (first separation attempt successful within 1 day, or death), difficult weaning (successful separation after 1 day to 1 week following initiation of weaning, or death), and prolonged weaning (successful separation still not obtained after 1 week following initiation of weaning, or death). Interestingly, this new classification convincingly confirmed that longer duration of ventilator weaning was associated with a higher mortality (Beduneau et al., 2017).

No large-scale study assessing the prevalence of WIPO according to the different weaning groups is currently available. Most descriptive studies reported the rate of WIPO at the origin of weaning failure in patients with difficult or prolonged ventilator weaning (Lemaire et al., 1988; Cabello et al., 2010; Liu et al., 2016; Goudelin et al., 2020). Typically, WIPO occurs rapidly after the initiation of the first SBT (Goudelin et al., 2020), but it may also develop after several SBTs or early after the first extubation in the subgroup of patients who are at highest risk (e.g., severe heart disease, COPD) (Lemaire et al., 1988). Weaning failure due to respiratory or neurologic causes typically develops later after extubation.

#### Patients at risk of WIPO

WIPO accounts for nearly 60% of weaning failure in patients with COPD (OR: 8.7 [2.0–37.3]), heart disease (OR: 4.5 [1.4–14.1]), or obesity (OR: 3.6 [1.2–12.6]) (Liu et al., 2016). Indeed, hemodynamic changes induced by SBT are amplified in patients with left heart disease (e.g., coronary artery disease, heart failure with reduced or preserved LV ejection fraction) or with COPD (Teboul, 2014). Cardiac ischemia may occur in patients with pre-existing coronary artery disease due to the marked increase in myocardial oxygen demand during SBT. Nevertheless, a recent study suggests that it is neither associated with WIPO nor with weaning outcome in difficult-to-wean patients (Bedet et al., 2019). Although LV systolic dysfunction is frequently identified using echocardiography in patients who fail SBT (Caille et al., 2010), LV ejection fraction remains stable during weaning as opposed to LV diastolic function which deteriorates with increased filling pressure (Caille et al., 2010; Moschietto et al., 2012; Goudelin et al., 2020). Accordingly, patients with LV diastolic dysfunction are at high risk of developing WIPO. Since they have a reduced diastolic and chronotropic reserve, and an abnormal ventriculo-arterial coupling (Borlaug and Paulus, 2011), they are particularly susceptible to any abrupt increase in LV afterload, such as that induced by acute hypertension associated with increased adrenergic tone during SBT. Importantly, occult cardiovascular insufficiency may also be a primary cause of weaning failure in critically ill patients with previously unrecognized left heart disease (Pinsky, 2000).

COPD patients are prone to develop WIPO for several reasons. First, the acute change of LV loading condition induced by SBT is exacerbated, since the hyperinflation and increased airway resistance during spontaneous ventilation require large negative inspiratory pressure to generate adequate tidal volumes (Lemaire et al., 1988; Perren and Brochard, 2013). Second, RV dilatation secondary to increased venous return restricts the LV within the stiff pericardial space due to ventricular interaction (Lemaire et al., 1988). Third, these patients have frequently associated left heart disease (Lemaire et al., 1988; Jubran et al., 1998). Jubran et al. (1998) showed that COPD patients who failed weaning exhibited progressive fall in mixed venous oxygen saturation (i.e., increased oxygen extraction), reflecting underlying cardiac decompensation.

#### Types of SBT

In the WIND study, the 2,904 SBTs performed by physicians were almost equally distributed between T-piece ventilation (49.9%) and low pressure ventilation trials (47.5%), while other types of SBTs were seldom (Beduneau et al., 2017). In a physiologic meta-analysis, Sklar et al. (2017) showed that SBT using pressure support reduces respiratory effort compared with T-piece, and that T-piece best reflects breathing efforts after extubation. In difficult-to-wean patients with a failed T-piece trial and a cardiac disease and/or COPD, a new T-piece trial failed in 11/14 patients (79%) due to LV failure (PAOP >18 mmHg), whereas SBT using pressure support ventilation with positive or zero end-expiratory pressure failed in only 3/14 (21%) and 6/14 (43%) patients, respectively (Cabello et al., 2010). Interestingly, mean PAOP was higher during T-piece than during SBT using pressure support, and exceeded 18 mmHg in 80% of patients with cardiac disease and in 50% of patients with COPD (Cabello et al., 2010). Overall, these results suggest that T-piece trial induces a more pronounced raise of LV filling pressure and of SBT failure due to WIPO in high-risk patients, when compared with pressure support trials. In addition, successful T-piece trial in these patients could better predict extubation success since this type of SBT best reflects physiological conditions during spontaneous breathing (Sklar et al., 2017). Similarly, in consecutive ICU patients ventilated for more than 24 h, a short (30 min) SBT using pressure support was associated with a higher risk of post-extubation respiratory failure and subsequent delayed extubation than a longer (60–120 min) T-piece trial (Subira et al., 2019).

Overall, T-piece trial elicits the greatest burden on respiratory and cardiocirculatory function during SBT. Since patients at high risk of WIPO require a higher threshold for extubation, a successful T-piece trial appears to sensitively assess their ability to tolerate fully unsupported spontaneous breathing (Fructos-Vivar and Esteban, 2014; Kuhn et al., 2016).

### Mechanisms of WIPO and tailored therapy

#### Illustration of underlying mechanisms using echocardiography monitoring

Excessive rise in LV filling pressure during SBT in patients who develop WIPO is multifactorial. Although the leading mechanism at the origin of WIPO may vary, positive fluid balance frequently associated with hypervolemia is common (Liu et al., 2016; Goudelin et al., 2020). Typically, WIPO develops shortly after the disconnection from the ventilator, with PAOP increasing within 10 min and even 5 min after the initiation of the T-tube trial (Lemaire et al., 1988). These clinical findings are in keeping with the prominent role of deleterious cardiac loading conditions induced by SBT. In high-risk patients monitored using echocardiography during SBT, Goudelin et al. (2020) have shown that WIPO was associated with the conjunction of excessive fluid balance and elevated LV filling pressure. In 117 unselected patients who were hemodynamically monitored during their first SBT, Caille et al. (2010) showed that those who failed exhibited a lower median LV ejection fraction and higher median E/E′ ratio (reflecting LV filling pressure) than their counterparts (36% [27–55] vs. 51% [43–55]: *p* = 0.04 and 7.0 [5.0–9.2] vs. 5.6 [5.2–6.3]: *p* = 0.04, respectively). Similarly, Ait-Oufella et al. (2007) reported that the increase in LV filling pressure (mitral E/A and E deceleration time) during SBT was significantly more pronounced in the subset of patients with LV systolic dysfunction. Finally, Papanikolaou et al. (2011) convincingly showed that the proportion of weaning failure was higher in patients with LV diastolic dysfunction based on mitral Doppler assessment, especially when of high grades (80% with grade 2 or 3 vs. 57% with grade 1 and 35% without LV diastolic dysfunction). In a recent meta-analysis, weaning failure was associated with echocardiography parameters indicating worse LV diastolic function (mitral Doppler E and E′ maximal velocity) and increased LV filling pressure (E/E’ ratio) (Sanfilippo et al., 2021).

**FIGURE 1:**
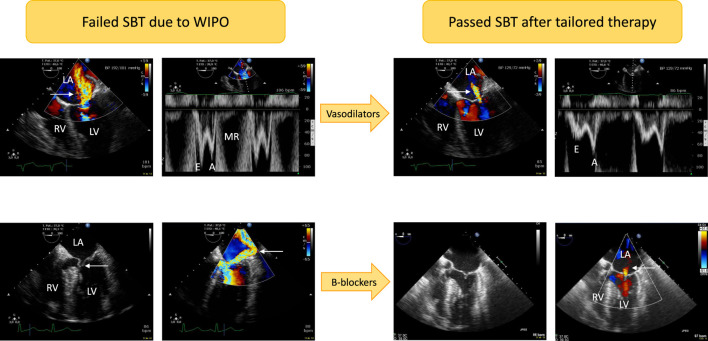
Illustrative cases of acute severe mitral regurgitation induced by abrupt changes in left ventricular loading conditions and increased adrenergic tone during a T-piece trial. Patients developed severe weaning-induced pulmonary edema and were promptly reconnected to the ventilator to be assessed using transesophageal echocardiography. In the first patient (upper panels) with acute hypertension (192/101 mmHg), a massive functional mitral regurgitation (central jet on color Doppler mapping, upper left panel, arrow) with markedly elevated mitral Doppler velocities (upper left middle panel; E wave maximal velocity: 1 m/s) was evidenced. Nitrates were administered intravenously as sequential boli to rapidly normalize blood pressure (129/72 mmHg). This allowed to dramatically reduce the volume of mitral regurgitation as reflected by a marked decrease of color Doppler jet area (upper right middle panel, arrow) as well as mitral Doppler velocities (upper right panel; maximal E wave velocity: 0.5 m/s). In the second patient (lower panels), echocardiography depicted a dynamic obstruction of left ventricular outflow tract by an anterior motion of the mitral valve (lower left panel, arrow). This resulted in high end-systolic pressure gradient and associated massive eccentric mitral regurgitation (lower middle left panel, arrow). Beta-blockers were successfully used: both the dynamic left ventricular outflow tract obstruction and mitral regurgitation fully resolved as depicted by two-dimensional imaging (lower middle right panel; end-systole) and color Doppler mapping which only disclosed a residual trivial central mitral regurgitation (lower right panel, arrow). Abbreviations: LA, left atrium; LV, left ventricle; RV, right ventricle; MR: mitral regurgitation.

Mitral regurgitation (MR) may participate in WIPO. Three mechanisms of MR may be distinguished during SBT: worsened functional MR, restrictive ischemic MR, eccentric MR secondary to a systolic anterior motion of the mitral valve with LV outflow tract obstruction (Figure 1). Abrupt SBT-induced changes in LV loading conditions (especially increased LV afterload) may result in acute worsening of functional MR and participate in WIPO (Goudelin et al., 2020). Gerbaud et al. (2012) showed that the area of mitral regurgitant jet during color Doppler mapping significantly increased at the end of SBT, especially in patients who failed, reflecting worsened MR volume. Increased work of breathing during SBT can induce ischemic MR with subsequent WIPO. Similarly, patients with ischemic heart disease and restrictive MR can develop acute pulmonary edema during an exercise stress test, with a significant increase of MR volume (Piérard and Lancellotti, 2004). Finally, dynamic LV outflow tract obstruction by an anterior systolic motion of the mitral valve secondary to increased sympathetic drive during SBT may result in acute and severe eccentric MR with associated WIPO (Goudelin et al., 2020). Importantly, right heart catheterization is unable to identify this mechanism of WIPO since the hemodynamic profile is typically characterized by markedly elevated PAOP, low cardiac index, and pulmonary hypertension which is consistent with decompensated heart failure (Adamopoulos et al., 2005).

Echocardiography study during SBT in COPD patients are scarce. The prevalence of elevated PAOP in these patients is high (Lemaire et al., 1988; Cabello et al., 2010). In patients with RV dilatation, Ait-Oufella et al. (2007) reported a significant increase of mitral Doppler E/A ratio and shortened E wave deceleration time during SBT, which suggested an increase of LV filling pressure potentially due to marked ventricular interaction. This mechanism may be operant in COPD patients who develop WIPO during SBT, especially in the presence of associated left heart disease.

#### Prediction of WIPO

Passive leg raising before SBT has been proposed to predict WIPO (Dres et al., 2015), as well as the increase of central venous pressure during SBT (Dubo et al., 2019). After a failed SBT due to WIPO, the shift from a negative to a positive passive leg raising after fluid removal likely predicts the success of following SBT (Liu et al., 2016).

Echocardiography is of major interest to early identify ventilated patients who are at high-risk of developing WIPO, before initiating SBT (Mayo et al., 2016). This population can be empirically defined as ventilated patients exhibiting LV systolic dysfunction (LV ejection fraction <40%), and/or severe LV diastolic dysfunction (grade 2 or 3) (Nagueh et al., 2016), significant valvulopathy (especially MR), obstructive cardiomyopathy irrespective of the presence of associated MR, preload-independency irrespective of the dynamic index used under mechanical ventilation, and RV dilatation with systemic venous congestion whether it is associated with underlying chronic respiratory insufficiency and severe pulmonary hypertension, or not.

During SBT, Moschietto et al. (2012) proposed a E/E′ threshold value of 14.5 to predict WIPO, with a sensitivity of 75% and a specificity of 96%. In contrast, low mitral E wave velocity (<0.6 m/s) at baseline without significant increase during SBT predicted a successful T-piece trial in all our patients at high-risk of WIPO (Goudelin et al., 2020). During SBT, the absence of 1) significant increase of E/E’, 2) substantial augmentation of systolic pulmonary artery pressure, 3) SBT-induced MR or MR worsening, 4) relevant regional wall motion abnormality or new-onset LV systolic dysfunction in an asymptomatic patient allows considering extubation with a low risk of failure (Vignon, 2018).

#### Guide to personalized therapy

Since diuretics reduce circulating blood volume and may also reduce airway edema and resistance, they constitute the mainstay of WIPO management (Lemaire et al., 1988; Perren and Brochard, 2013; Teboul, 2014). In a randomized controlled trial, fluid management strategy guided by circulating BNP level for ventilator weaning resulted in increased diuretics use, more negative fluid balance and shorter duration of mechanical ventilation, especially in patients with LV dysfunction (Mekontso-Dessap et al., 2012). Nevertheless, as mentioned earlier, excessive LV filling pressure during SBT may be related to other mechanisms than hypervolemia. Although fluid overload is a key determinant for developing WIPO, systematic fluid removal in all patients at risk could have deleterious effects (e.g., acute renal failure, hypokalemia and rhythm disorders). Alternatively, we previously showed in high-risk patients undergoing SBT that echocardiography efficiently guides tailored therapy according to the underlying mechanism leading to WIPO (e.g., volume or pressure overload, severe MR, dynamic obstruction to LV ejection), which allowed successful extubation in all patients who failed a first SBT after an average of 2.5 days (Goudelin et al., 2020).

**FIGURE 2:**
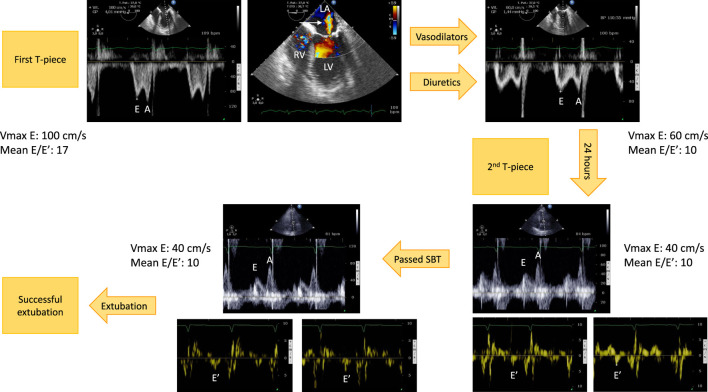
Example of hemodynamic monitoring using echocardiography in a patient with left ventricular systolic dysfunction secondary to an ischemic heart disease who developed a weaning-induced pulmonary edema during the first T-piece trial which induced high blood pressure. Immediately after having reconnected the patient to the ventilator, a transesophageal echocardiography was performed. Mitral Doppler depicted elevated E wave velocity and mean E/E′ ratio, consistent with increased left ventricular filling pressure (upper left panel). There was only a mild functional mitral regurgitation (upper middle panel, arrow). Treatment associated short half-life sedative, ACE inhibitors and diuretics. With normalization of blood pressure and fluid removal, the mitral Doppler profile remained unchanged (E/A ratio ≈1), but velocities significantly decreased as well as mean E/E′ ratio (upper right panel). This allowed to perform a second T-piece trial 24 h later. Immediately before the procedure, mitral E wave velocity remained low, E/A ratio was <1, and mean E/E′ remained stable, denoting the absence of new rise in left ventricular filling pressure (3 lower right panels). The second spontaneous breathing trial was clinically successful. Echocardiography at the end of the 30-min T-piece trial depicted similar mitral Doppler velocity values, hence left ventricular filling pressure (3 lower left panels). The patient was then extubated successfully without any change of ongoing treatment. Abbreviations: LA, left atrium; LV, left ventricle; RV, right ventricle.

Vasodilators can preferably be used in hypertensive patients who develop WIPO secondary to SBT-induced high blood pressure, including initial intravenous boluses to rapidly decrease systolic arterial pressure, hence LV afterload (Goudelin et al., 2020). Nitrates associate beneficial pharmacological effects in this clinical setting: reduction of central blood volume (venous dilatation), reduction of LV afterload (arterial dilatation, decrease of systolic blood pressure), and coronary vasodilatation (increased myocardial perfusion to respond to enhanced myocardial oxygen consumption) (Figure 1). In 12 difficult-to-wean COPD patients who failed at least 3 SBTs, the administration of nitrates avoided the rise of PAOP and of systolic arterial pressure during a 2-h T-piece trial and allowed successful extubation (Routsi et al., 2010). We also reported the successful use of nitrates and ACE inhibitors in controlling high blood pressure and allowing successful extubation after a first failed SBT due to WIPO (Goudelin et al., 2020). In this case, echocardiography showed that SBT-induced variation of maximal E wave velocity (reflecting LV filling pressure) significantly decreased when compared to initial failed T-piece trial and was similar to that observed in patients who successfully passed the first SBT. Finally, vasodilators such as ACE inhibitors may be proposed in association to diuretics in ventilated patients at risk of WIPO due to underlying severe LV diastolic dysfunction with elevated LV filling pressure identified by echocardiography, to prevent SBT failure (Figure 2).

Beta-blockers can also be advantageously used in the specific setting of LV outflow tract obstruction associated with an anterior systolic motion of the mitral valve and eccentric MR (Adamopoulos et al., 2005; Goudelin et al., 2020). The adrenergic response to SBT promotes dynamic obstruction to LV ejection, especially when associated to an abrupt rise in systolic arterial pressure. To counteract these deleterious effects, reduction of high blood pressure and negative inotropic and chronotropic drugs such as beta-blockers allow to dramatically reduce both the pressure gradient at the level of LV outflow tract and associated volume of MR jet (Figure 1).

The use of inotropes in patients developing WIPO appears counterintuitive since LV contractility does not seem to be hampered by SBT, even in patients with previous LV systolic dysfunction (Caille et al., 2010; Sanfilippo et al., 2021). Moreover, additional catecholamine load may further increase myocardial oxygen consumption and precipitate dynamic LV outflow tract obstruction. Nevertheless, inodilators such as Levosimendan have been successfully used in patients with LV systolic dysfunction who failed a first SBT. After a 24-h infusion of Levosimendan in 11 ventilated patients, echocardiography depicted improved LV systolic and diastolic function and mitigated the increase of E/E’—hence LV filling pressure—during the second SBT, which was successful in 9 of them (82%) (Kaltsi et al., 2019). In a small size sequential study evaluating difficult-to-wean COPD patients with right heart catheterization during SBT, Ouanes-Besbes et al. (2011) showed that Levosimendan provided a greater reduction of PAOP increase induced by the T-piece trial, when compared to Dobutamine.

Finally, myocardial ischemia induced by SBT is seldom responsible for WIPO (Bedet et al., 2019). Nevertheless, SBT-induced myocardial ischemia may result in an acute restrictive MR which rapidly translate in WIPO. Even less frequently, coronary revascularization is required to allow successful extubation in patients who develop WIPO secondary to SBT-induced acute myocardial ischemia (Demoule et al., 2004; Carrié et al., 2011; Bedet et al., 2019).

#### Incorporating echocardiography monitoring in the weaning process

**FIGURE 3:**
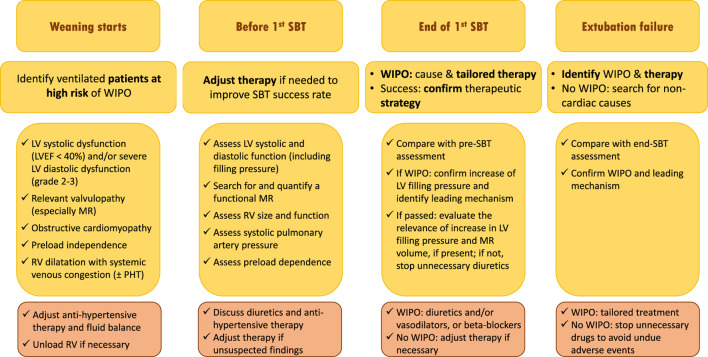
Proposed strategy of use of echocardiography monitoring in ventilated patients who are ready to be weaned from the ventilator and potentially at risk of weaning failure of cardiac origin (see text for details). Abbreviations: SBT, spontaneous breathing trial; WIPO, weaning-induced pulmonary edema; LV, left ventricle; EF, ejection fraction; MR, mitral regurgitation; RV, right ventricle; PHT, pulmonary hypertension.

Since the level of evidence of WIPO treatment is low for non-diuretics drugs and only based on physiology and small sample size, non-randomized interventional studies, a personalized approach in high-risk patient based on echocardiography monitoring is attractive to tailor therapy (Vignon, 2018) (Figure 3). Patients at risk of weaning failure and WIPO (i.e., >65 years old, medical history of cardiac or respiratory disease, obesity) should first be assessed by echocardiography at the beginning of their weaning process. This will allow to confirm the risk of WIPO based on the identification of underlying cardiac disease and evaluation of LV filling pressure, and to prevent or reduce LV overload hence positive hydric balance. Echocardiography should then be performed before initiating the first SBT, to allow the correction of unsuspected abnormal findings which could preclude the success of weaning (e.g., persisting LV overload, significant functional MR). At the end of SBT, echocardiography assessment should be compared to that performed at baseline to ascertain that LV filling pressure fails to significantly increase and that MR severity is stable, if present at baseline. Asymptomatic yet significant SBT-induced LV filling pressure in patients who succeed their first SBT suggests adjusting therapy to prevent subsequent extubation failure due to WIPO. In case of failed SBT, echocardiography is immediately performed to best guide tailored management according to the leading mechanism of WIPO, before or after reconnection of the patient to the ventilator. In contrast, the absence of significant LV overload under invasive mechanical ventilation or during SBT in asymptomatic patients will avoid to uniformly administer unnecessary treatment with potential side effects (e.g., diuretics). The herein proposed strategy to improve ventilator weaning in patients at high risk of WIPO (Figure 3), remains to be validated by prospective interventional studies.

## Conclusion

The transition from positive-pressure ventilation to spontaneous breathing may elicit an excessive burden on both the respiratory and cardiovascular systems, and result in WIPO. The population at risk is broad and occult cardiac disease may be revealed at the time of SBT. Although positive fluid balance frequently participates in WIPO, alternative leading mechanisms may also be operant. Echocardiography is best suited for identifying high-risk patients, guiding early strategy to reduce LV overload, depicting the origin of WIPO, tailoring individual management, to ultimately increase the success rate of further SBTs and extubation. The effect of such strategy on patient-centered outcomes needs to be evaluated in a large population of patients at high risk of weaning failure of cardiac origin.
